# Ficolin B in Diabetic Kidney Disease in a Mouse Model of Type 1 Diabetes

**DOI:** 10.1155/2015/653260

**Published:** 2015-08-03

**Authors:** Charlotte Berg Holt, Jakob Appel Østergaard, Esben Axelgaard, Gitte Krogh Nielsen, Yuichi Endo, Steffen Thiel, Troels Krarup Hansen

**Affiliations:** ^1^Department of Endocrinology and Internal Medicine, Aarhus University Hospital, 8000 Aarhus, Denmark; ^2^Department of Biomedicine, Aarhus University, 8000 Aarhus, Denmark; ^3^The Danish Diabetes Academy, Sdr. Boulevard 29, 5000 Odense, Denmark; ^4^Department of Immunology, Fukushima Medical University School of Medicine, Fukushima 960-1295, Japan

## Abstract

*Background.* The innate immune system may have adverse effects in diabetes and cardiovascular disease. The complement system seems to play a key role through erroneous complement activation via hyperglycaemia-induced neoepitopes. Recently mannan-binding lectin (MBL) was shown to worsen diabetic kidney changes. We hypothesize that mouse ficolin B exerts detrimental effects in the diabetic kidney as seen for MBL. *Methods.* We induced diabetes with streptozotocin in female wild-type mice and ficolin B knockout mice and included two similar nondiabetic groups. Renal hypertrophy and excretion of urinary albumin and creatinine were quantified to assess diabetic kidney damage. *Results.* In the wild-type groups, the kidney weighed 24% more in the diabetic mice compared to the controls. The diabetes-induced increase in kidney weight was 29% in the ficolin B knockout mice, that is, equal to wild-type animals (two-way ANOVA, *P* = 0.60). In the wild-type mice the albumin-to-creatinine ratio (ACR) was 32.5 mg/g higher in the diabetic mice compared to the controls. The difference was 62.5 mg/g in the ficolin B knockout mice, but this was not significantly different from the wild-type animals (two-way ANOVA, *P* = 0.21). *Conclusions.* In conclusion, the diabetes-induced effects on kidney weight and ACR were not modified by the presence or absence of ficolin B.

## 1. Introduction

Diabetes is the most frequent cause of end-stage renal disease in industrialised countries [1, 2]. Clinically, diabetic nephropathy is characterized by the development of albuminuria and a subsequent decline in glomerular filtration rate. This severe complication significantly influences the risk of cardiovascular disease as well as mortality and quality of life [3, 4]. At present the most important identified risk factors are diabetes duration, arterial blood pressure, and glycaemic regulation [5]. As evident from an increasing incidence of affected patients, however, there is still a great need for new strategies in the treatment and prevention of diabetic nephropathy.

Clear evidence indicates that the pathogenesis of diabetic nephropathy is multifactorial and triggered by a complex series of pathophysiological events [6]. Inflammation has emerged as a key factor in the development of diabetic nephropathy [7]. The inflammatory response in diabetes is highly complex involving proinflammatory cytokines and chemokines, for example, IL1, IL6, IL8, TNF, and NF*κ*B [8]. The impact of complement activation on the diabetic kidney may well, in part, be mediated through induction of cytokine response and inflammation [9–11].

Several studies have linked diabetic late-complications to the complement system of the innate immune system [12, 13]. The complement system plays a crucial role in recognition and clearance of infectious microbes and the system forms a link between innate and adaptive immunity [14]. The complement system functions as an enzymatic cascade. The activation of the complement system results in the release of multiple inflammatory signaling molecules. Ultimately complement activation leads to the formation of pore-forming membrane attack complexes (MACs) that are inserted in the cell membranes to mediate lyses of the cell through osmotic stress.

However, in mammalian cells, it has been shown that sublytic amounts of MAC can increase production of IL-8 and monocyte chemoattractant protein 1 dependent on NF*κ*B nuclear translocation [15]. Furthermore, MACs are shown to have a mitogenic effect and cause release of basic fibroblast growth factor and platelet-derived growth factor from endothelial cells leading to fibrosis in neighboring cells including glomerular mesangial cells [16, 17]. The latter effects of MAC may explain the link between complement and diabetic kidney damage.

Three activations pathways exist: the classical, the alternative, and the lectin pathway. The present paper focuses on the lectin pathway, in which at least five soluble pattern-recognition molecules are characterized that may activate the complement system, that is, mannan-binding lectin (MBL), H-ficolin, L-ficolin, M-ficolin, and collectin-K1 [18]. The three ficolins utilize a fibrinogen-like domain that binds, for example, N-acetylglucosamine, N-acetylgalactosamine, and N-acetyl-neuraminic acid, whereas MBL and CL-K1 have a carbohydrate recognition domain and through this bind specifically to patterns of monosaccharides. A very recent publication reports a close association between ficolin and diabetic nephropathy in patients with type 1 diabetes [19]. The observational design of the study, however, limits its ability to study a cause-effect relationship. When ficolins bind they all initiate activation of associated serine proteases (MBL associated serine proteases, MASPs), which subsequently cleave the complement factors, C2 and C4, leading to further complement activation [20, 21]. Eventually, complement activation leads to the formation of MACs causing cell lyses or induction of fibrosis [22].

The balance between activation and inhibition of the complement cascade is tightly controlled by regulatory proteins in order to prevent damage of healthy host cells. In diabetes, inappropriate effects of the complement system may be present as glycation-induced dysfunction of the complement inhibitory mechanism and consequently overactivation of the system is indicated [23–25]. It is speculated that diabetic patients are exposed to uncontrolled complement attack partly due to altered molecular patterns on the cell surfaces as a consequence of high blood glucose [24, 25]. Most significantly, an association is seen between diabetic nephropathy and the lectin pathway [26–28]. We have previously demonstrated direct cause-effect relationship between presence of MBL and worsening of kidney injury in a mouse model of diabetic nephropathy [29, 30]. We speculate that ficolins also exert detrimental effects in diabetes similar to MBL through activation of the lectin pathway as indicated in patients with type 1 diabetes [19].

This study aimed to investigate the impact of ficolin B (the orthologue to human M-ficolin) on the development of diabetic nephropathy in a mouse model of type 1 diabetes.

## 2. Materials and Methods

### 2.1. Animals

We used 11-week-old, female ficolin B knockout mice and age-matched, female C57BL/6J BomTac wild-type mice (Taconic, Ry, Denmark). The knockout ficolin B model was backcrossed more than 10 generations to a C57BL/6J BomTac genetic background (own breeding) [31].

In each cage there were three to eight mice and they had free access to tap water and standard chow (Altromin number 1324; Lage, Germany). The environment was stable with a 12-hour light-dark cycle, temperature at 21 ± 1°C, and humidity of 55 ± 5%. The study complied with the regulations for care and use of laboratory animals.

The ficolin B knockout mice and the wild-type mice were randomized into a diabetic and nondiabetic group; thus four groups were made: (1) diabetic knockout mice (*n* = 6), (2) nondiabetic knockout mice (*n* = 7), (3) diabetic wild-type mice (*n* = 11), and (4) nondiabetic wild-type mice (*n* = 11).

Diabetes was induced by intraperitoneal injections of streptozotocin (STZ) dissolved in a cold 10 mM citrate buffer (doses of 55 mg/kg body weight, Sigma Aldrich, St Louis, Mo, USA) on five consecutive days [32]. The animals were fasting 4 hours prior to injection. If the blood glucose did not rise sufficiently two more injections were given. The diabetic mice received the exact same amount of STZ. Controls were injected with citrate buffer only. The 18-week experiment was initiated when the mice were classified as diabetic (blood glucose > 15 mM).

Animals with more than 15% sustained weight loss, signs of illness, or persistent ketonuria were excluded from the study. Body weight and blood glucose were measured weekly. Blood glucose was measured from tail vein by Contour (Bayer Diabetes Care, Kgs. Lyngby, Denmark). With Combur^5^ Test D strip (Roche Diagnostics GmbH, Mannheim, Germany) urine was tested for ketone bodies. Two mice from each diabetic group were excluded because of insufficient increase in blood glucose levels. Furthermore, two mice from the diabetic knockout group were excluded because of weight loss > 15% of body weight. The excluded mice were not included in the number of animals per group indicated above.

### 2.2. Collection of Samples

Spot urine was collected in eppendorf tubes on five consecutive days prior to sacrifice of the animals. The blood samples were drawn from under the tongue at baseline and from the retroorbital venous plexus at study end and collected in potassium EDTA tubes (Sarstedt, Nümbrecht, Germany). Both urine and blood samples were stored at −80°C until analysed.

The animals were anesthetized by an intraperitoneal dose of ketamine at 0.5 mg/g body weight and xylazine at 0.2 mg/g body weight (Ketaminol 4 Vet and Narcoxyl Vet, resp., Intervet, Skovlunde, Denmark). The kidneys were dissected and weighed, after which the mice were sacrificed. The measures of left kidney weight were used in the analyses.

### 2.3. Albumin-to-Creatinine Ratio (ACR)

Urinary albumin excretion was determined by Mouse Albumin ELISA quantification Kit (Bethyl laboratories, Inc., Montgomery, TX, USA) according to the manufactory's instruction.

Urine creatinine was measured by isocratic high-performance liquid chromatography (HPLC) on a Zorbax SCX300 column (Agilent, USA) using a slight modification of a method first reported by Yuen et al. [33]. In brief, 5 *μ*L urine was added to 100 *μ*L acetonitrile containing 0.5% acetic acid and vortexed for 15 seconds to extract the creatinine. After 15 min of −20°C storage and centrifugation the supernatants were evaporated and then reconstituted with 25 *μ*L 5 mM sodium acetate, pH 4.1. The samples were centrifuged for 10 min at 3000 rpm. Duplicate samples (10 ul each) were fractionated on a 50 mm × 2.1 mm Zorbax SCX300 column with an in-front SCX guard column. Isocratic HPLC was performed at a flow rate of 1 mL/min, and UV absorbance was monitored at 225 nm. A standard curve was created by including a 2-fold dilution series of creatinine anhydrous (Sigma Aldrich). All aqueous solutions were filtered through a 0.22-micron filter before use.

### 2.4. Statistics

This study was designed with two independent factors; diabetes/nondiabetes and knockout/wild-type and thus analysed by two-way ANOVA for normal distributed variable with equal variance. The main focus of interest was the interaction between the diabetic factor and the knockout factor; that is, does ficolin B modify the effects of diabetes on the effect parameters? If no interaction was found, the independent effects of diabetes and ficolin B on the kidney were estimated. For pairwise comparison, normal distributed data was tested with Student's *t*-test, whereas otherwise the Wilcoxon Mann-Whitney rank sum test was used. *P* values below 5% were considered as statistically significant. Data are given as mean (95% confidence interval (CI)) unless else is stated. All statistical analyses were performed using STATA version 12.

## 3. Results

### 3.1. Body Weight and Blood Glucose

**Figure 1:**
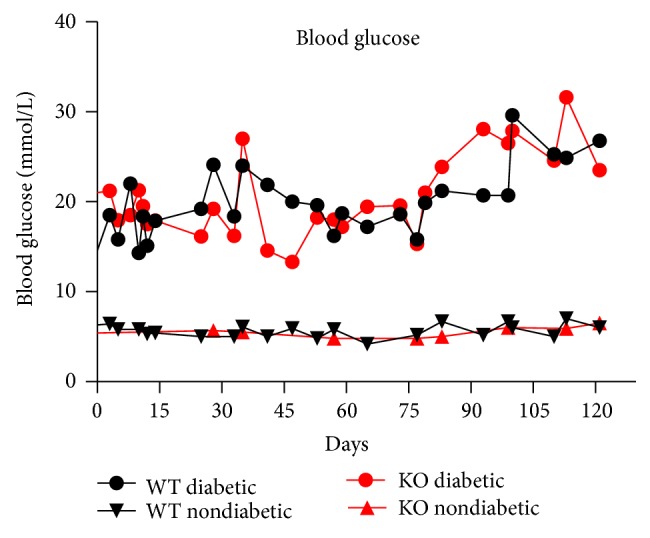
Mean blood glucose level in mmol/L in each of the four groups over time.

At baseline, the knockout mice on average weighed 20.0 g, which was slightly less than the wild type mice, 20.8 g (*P* = 0.04). No difference was found between the two diabetic groups or between the two nondiabetic groups (Table 1). After 18 weeks an expected difference in body weight was observed between the diabetic and the nondiabetic mice independently of knockout status (*P* < 0.001). The nondiabetic mice weighed 3.2 g (CI: 2.0 g–4.3 g) more than the diabetic mice. Furthermore the diabetic knockout mice were significantly smaller than the diabetic wild type (*P* < 0.05). As presented in Table 1, blood glucose, estimated as area under the curve (AUC), did not differ between the two diabetic groups (*P* = 0.69) or between the two nondiabetic groups (*P* = 0.13). The overall fluctuations in blood glucose in each group are depicted in Figure 1.

### 3.2. Kidney Weight

**Figure 2:**
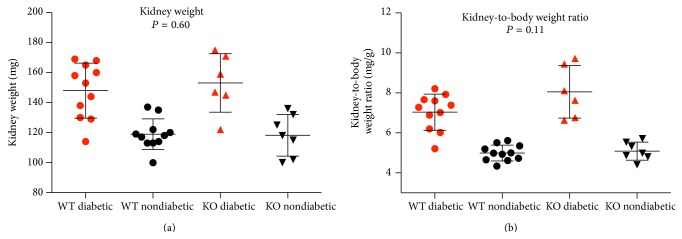
Kidney weight (a) and kidney weight per body weight (b) depicted for each of the four groups. The black error bars are illustrating mean ± standard deviation for each group. WT: wild-type; KO: ficolin B knockout.

The kidney weight was equally increased in diabetic wild-type mice, 24% (CI: 13%–36%), and in the diabetic knockout mice, 29% (CI: 12%–47%), compared to the respective control groups (Figure 2(a)). No interaction between knockout and diabetes was found (*P* = 0.60), indicating that wild-type and knockout mice develop the same degree of diabetes-induced renal hypertrophy. The considerable body weight difference between the two diabetic groups at study end indicated that the kidney weight was to be normalised to the body weight. This is illustrated in Figure 2(b). Ficolin B did not modify the diabetes-induced increase in kidney weight when testing for interaction (*P* = 0.11). Furthermore, no significant statistical difference was found in kidney weight per body weight between the diabetic wild-type, 1.95 mg/g, and the diabetic knockout, 2.89 mg/g (*P* = 0.09).

### 3.3. Albumin-to-Creatinine Ratio

**Figure 3:**
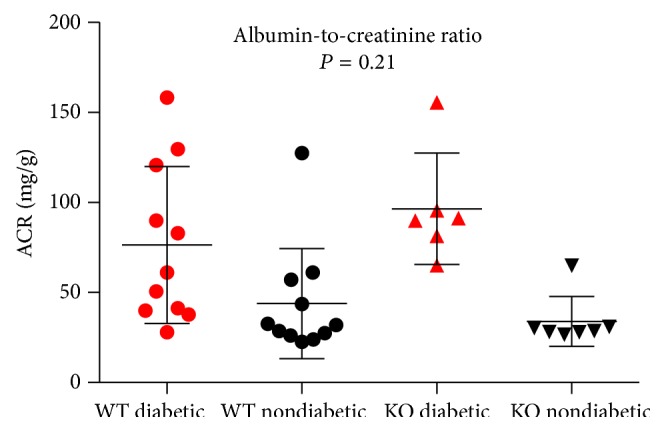
Albumin-to-creatinine ratio (mg/g) is illustrated for each of the four groups. The black error bars are illustrating mean ± standard deviation. WT: wild-type; KO: knockout.

The albumin-to-creatinine ration (ACR) was higher among the diabetic wild-type mice, 76 mg/g (CI: 50–103 mg/g), compared to the nondiabetic wild-type mice, 44 mg/g (CI: 25–63 mg/g), *P* = 0.07. Similarly, the ACR of diabetic knockout mice was 96 mg/g (CI: 71–122 mg/g) compared to the nondiabetic knockout group, 34 mg/g (CI: 23–44 mg/g), *P* < 0.001. As depicted in Figure 3 no interaction was observed between diabetes and ficolin B knockout, *P* = 0.21.

## 4. Discussion

In the present study we found no association between diabetes-induced kidney changes and the presence of ficolin B. We conclude that ficolin B is not responsible for, or a crucial contributory factor in, the pathophysiology of diabetic nephropathy. In our study, the kidney weight and to some extent the ACR were altered by diabetes as expected. The diabetes-induced increase in kidney weight, measured by comparing the diabetic mice with the nondiabetic mice, was not statistically different between the wild-type and ficolin B knockout mice. In other words, there was no effect modification or interaction. The diabetes-induced increase in kidney weight was 24% in the wild-type mice and 29% in the ficolin B knockout mice. Taking the lower body weight of the knockout mice into account, the difference in renal hypertrophy was still insignificant when comparing the wild-type mice and the ficolin B knockout mice. Similarly, the diabetic change seen in ACR was not altered in the absence of ficolin B.

The experimental setup including four groups matched on age, body weight, and genetical background was a strength to the study, as the diabetes factor and the knockout factor were the only modulators of the outcome. The two diabetic groups and the two nondiabetic groups had comparable glucose levels. Most importantly both diabetic groups did reach and sustain blood glucose levels of above 15 mM. At study end, the body weight differed among groups, which impeded the analyses of the diabetic kidney damage, because the knockout mice appear to be more vulnerable to type 1 diabetes mellitus.

Our study provides important new information on the association between the lectin pathway and diabetic kidney damage. We are the first to investigate the role of ficolin B (which corresponds to ficolin M in human) in the inflammatory response of diabetic nephropathy. In mice with deficiency of MBL, the classical functional and physical renal changes normally seen in this experimental model of type 1 diabetes were modified [29, 30]. This supports a number of human studies [26–28, 34]. The fact that ficolin B does not appear to modulate diabetic effects on the kidney emphasizes the importance of MBL compared with ficolin B. Both MBL and ficolin B activate the lectin pathway of the complement system, but only deficiency of MBL has been shown to protect against diabetic kidney damage [29]. This indicates that the role of the lectin pathway in the development of diabetic nephropathy is complex and may depend on the specific carbohydrate-binding properties of MBL as previously described.

The function of other complement factors in the first parts of the lectin pathway (e.g., ficolin A and MASPs) in the pathology of diabetic kidney disease remains unknown and must be explored in further studies. One study indicates that ficolin A and ficolin B exert a cooperatively defensive role in destroying Streptococcus Pneumoniae, suggesting a synergetic immunological effect [35]. This emphasises the need for further investigations involving both mouse ficolins. In order to fully understand the involvement of the lectin pathway in the development of diabetic nephropathy, an additional parallel experiment with MASPs is of particular interest given that they represent the limiting downward step in the complement activation.

## 5. Conclusion

In conclusion, this study demonstrates that ficolin B does not modify the kidney weight and ACR in a type 1 diabetes mouse model. This indicates that the role of the lectin pathway in the development of diabetic nephropathy is specific and that hyperglycaemia-induced glycations on renal cells may be more prone to bind MBL than ficolin B.

## Figures and Tables

**Table 1:** Table illustrating mean (95% confidence intervals).

		Groups				Two-way ANOVA	Student's *t*-test	
		Wild-type diabetic *N* = 11	Wild-type nondiabetic *N* = 11	Knockout diabetic *N* = 6	Knockout nondiabetic *N* = 7	Interaction *P* (KO ∗ dia)	*P* diabetes (WT><KO)	*P* control (WT><KO)
Body weight (g)	Start	20.6 (19.8; 21.4)	21 (20.2; 21.8)	20.2 (19.5; 20.8)	19.8 (19.1; 20.4)	0.38	0.43	0.05
	End	21.1 (19.9; 22.4)	23.8 (23.0; 24.6)	19.1 (18.0; 20.3)	23.2 (22.3; 24.2)	0.22	0.05	0.34

Blood glucose (mmol/L)	Start	6.8 (6.5; 7.2)	6.4 (5.9; 6.9)	5.2 (4.6; 5.8)	5.7 (5.1; 6.4)	0.08	<0.001	0.12
	End	23.8 (20.8; 26.8)	5.5 (4.9; 6.1)	26.3 (20.1; 32.5)	6.5 (6.0; 7.1)	0.60	0.41	0.04
	AUC	2526 (2358; 2692)	765 (698; 833)	2581 (2367; 2795)	660 (628; 691)	0.25	0.69	0.13

Kidney weight (mg)	End	148.0 (136.8; 159.2)	118.9 (112.6; 125.2)	153.2 (137.0; 169.4)	118.3 (107.6; 129.0)	0.60	0.59	0.92

Albumin-to-creatinine ratio (mg/g)	End	76.3 (49.6; 103.1)	43.8 (25.1; 62.6)	96.4 (70.8; 122.1)	33.9 (23.3; 44.5)	0.21	0.34	0.44

Interaction *P* (KO *∗* dia): two-way ANOVA—the interaction between the knockout factor and the diabetic factor.

*P* Diabetes WT><KO: Student's *t*-test of the diabetic wild-type mice compared to diabetic ficolin B knockout mice.

*P* Control WT><KO: Student's *t*-test of the control wild-type mice compared to the control ficolin B knockout mice.

AUC: blood glucose measured as area under the curve (days*∗*mmol/L).
